# Vaping and Commitment Flu-B Infection Is a Deadly Combination for Spontaneous Pneumomediastinum

**DOI:** 10.1155/2021/9944491

**Published:** 2021-06-17

**Authors:** Md Didar Ul Alam, Khandakar Hussain, Samsone Garedew, Muhammad imtiaz

**Affiliations:** ^1^Department of Internal Medicine, Conemaugh Medical Memorial Center, Johnstown, PA, USA; ^2^Department of Hospital Medicine, Conemaugh Medical Memorial Center, Johnstown, PA, USA

## Abstract

Vaping or E-cigarettes were created to deliver nicotine-containing aerosol to users with a flavoring agent without agents such as tars, oxidant gases, and carbon monoxide smoke found in traditional tobacco cigarettes. The use of E-cigarettes is steadily increasing in the United States, especially among the young population. Electronic cigarettes seem capable of causing various injury patterns in the lungs, collectively called E-cigarettes or vaping-associated lung injury (EVALI). Spontaneous pneumomediastinum (SPM) is a rare finding in EVALI. Here, we report a case of spontaneous pneumomediastinum secondary to vaping in a young man with no past medical history except for daily vaping and a recent untreated influenza infection.

## 1. Introduction

Pneumomediastinum is defined as the presence of air or other gas in the mediastinum also known as mediastinal emphysema [1]. Pneumomediastinum can be categorized as spontaneous pneumomediastinum (SPM) and secondary pneumomediastinum. Secondary pneumomediastinum is caused by blunt or penetrating chest wall trauma, barotrauma from mechanical ventilation, underlying airway diseases, endobronchial, and esophageal perforation. Vaping or E-cigarettes gained popularity over the last few years. E-cigarettes containing nicotine and other substances vaporize or produce aerosols, which the users then inhale. Predisposing conditions or triggers are identified in some cases of pneumomediastinum in children. A respiratory illness that led to pneumomediastinum, especially during acute exaggeration with excessive coughing, i.e., in asthma and respiratory infections, is identified in children and adolescents [2–5]. The unique aspect was that the development of SPM was triggered by excessive coughing from untreated influenza infection in an otherwise healthy young man with concomitant lung injury from daily vaping.

## 2. Case Presentation

**Figure 1:**
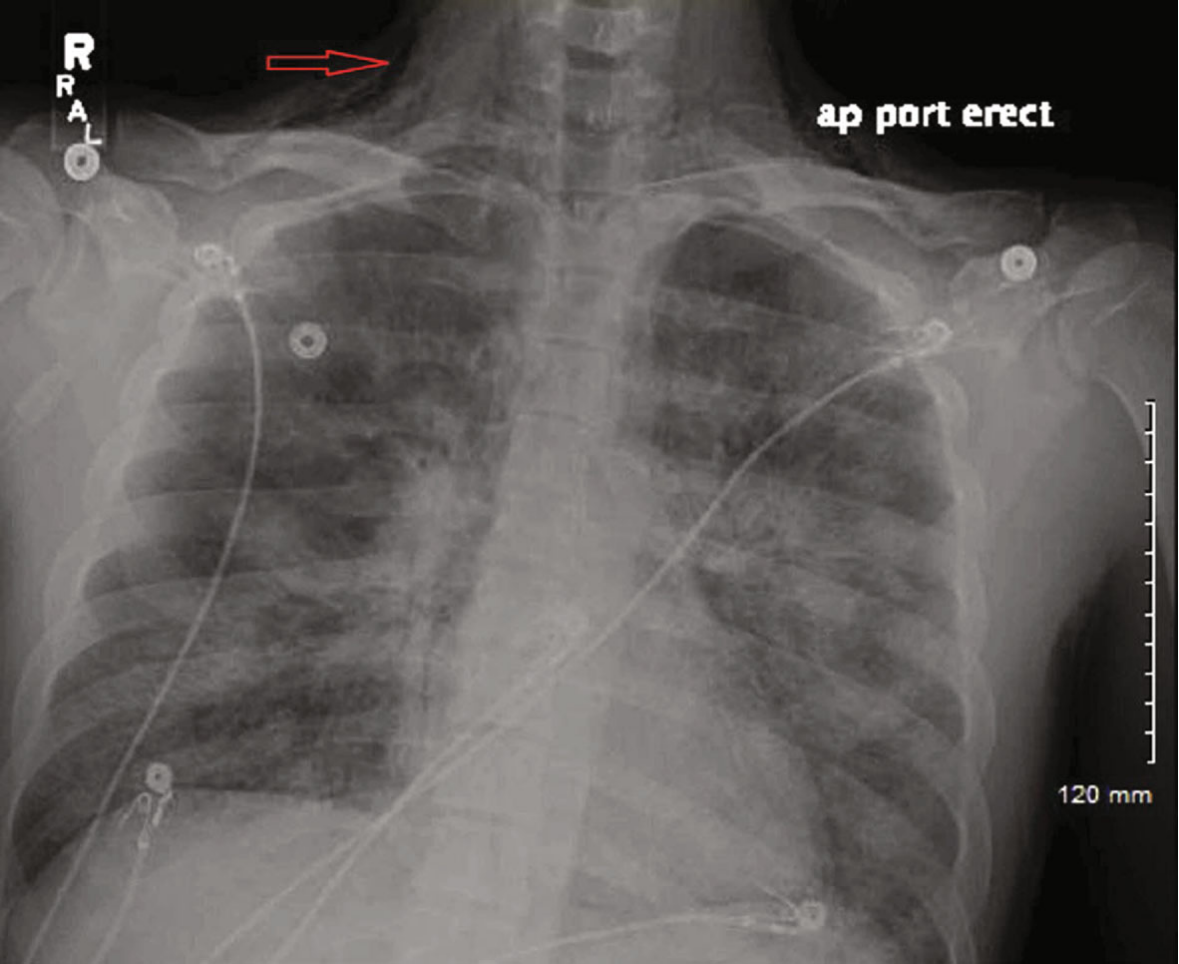
X-ray showing pneumomediastinum with subcutaneous emphysema (red arrow) extending to the cervical region with bilateral pneumonia.

A 22-year-old male was brought to the emergency department (ED) for cough and progressive shortness of breath for the last six days. His past medical history is unremarkable except for daily vaping. A week ago he also tested positive for influenza B when he had chills, fatigue, and sore throat. He did not take oseltamivir due to a late prescription from his primary care physician. He had low-grade fever with nausea and vomiting with fierce cough four days before presentation to ED. He also felt crepitus in the neck. He did not have any prior chest surgical history or any history of illicit drug abuse. In the ED, his temperature was 99.1 degrees Fahrenheit, blood pressure of 150/95 mmHg, heart rate of 136 beats/min, and a saturation of 91% on 3 L nasal cannula (NC) oxygen. On physical examination, subcutaneous emphysema was noted in the anterior cervical region. Blood work showed leukocytosis of 18,000/mL and a procalcitonin level of 0.21 ng/mL. Chest X-ray (CXR) showed pneumomediastinum with subcutaneous emphysema extending into the cervical region with bilateral pneumonia (Figure 1).

**Figure 2:**
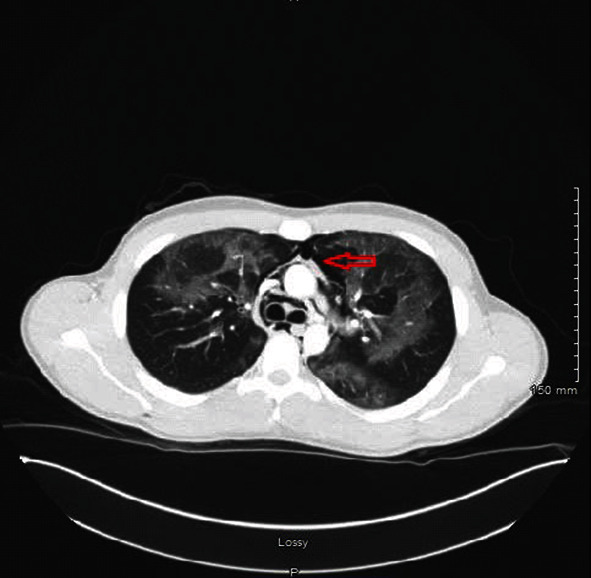
Computed tomography (CT) chest showing severe pneumomediastinum (red arrow) with diffuse pneumonitis.

Chest computed tomography (CT) showed severe pneumomediastinum with subcutaneous emphysema and diffuse pneumonitis (Figure 2).

**Figure 3:**
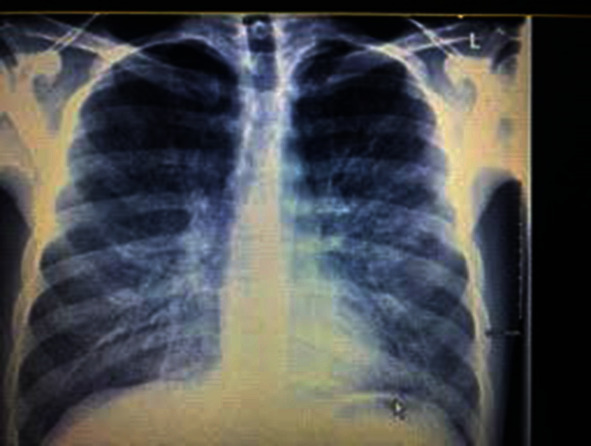
Improvement of pneumomediastinum and subcutaneous emphysema.

The patient was admitted to the medical floor. He was treated with supplemental 3 L NC supplemental oxygen, and oseltamivir and antibiotics were discontinued. A gastrografin esophagogram was done, and esophageal rupture was ruled out. Repeat CXR two days later showed improvement of pneumomediastinum and subcutaneous emphysema (Figure 3). He was counseled extensively against vaping and was discharged home on room air.

## 3. Discussion

SPM occurs due to air leaks through small alveolar ruptures to the surrounding bronchovascular sheath. Pneumomediastinum also could result from air leaks from the gastrointestinal tract (esophageal rupture) and respiratory tract [1, 6]. The proposed mechanism occurs due to differential pressure gradient that develops between the alveoli and lung interstitium. This pressure gradient can occur either by increasing the alveolar pressure (Valsalva maneuver) or by decreasing the interstitial pressure (increased work of breathing). This leads to alveolar rupture and release of air into a mediastinal space with lower pressure (Macklin effect) [7]. As the mean pressure in the mediastinum is always negative than the pressure in the pulmonary parenchyma, the free air tends to move centripetally along the vascular sheaths, facilitated by the pumping action of breathing. The air spreads to the hilum and into the mediastinum or through the loose mediastinal fascia to the subcutaneous tissues of the thorax, upper limbs, and neck. Recently, postulated mechanisms by which vaping can lead to acute lung injury include airway remodeling, macrophage activation, and direct injury of airway epithelium [8].

In most cases, the movement of air into the subcutaneous tissues prevents the buildup of pressure in the mediastinum. Occasionally, air leaks into the pericardial space, causing pneumopericardium [9, 10]. In extremely rare cases, pressure accumulates in the mediastinal cavity, causing pneumothorax or compression of adjacent intrathoracic structure leading to tension pneumomediastinum or tension pneumopericardium [11–14].

The classic triad of pneumomediastinum is retrosternal pleuritic pain (with exacerbation during deep inspiration), subcutaneous emphysema, and dyspnea. Chest radiography including the cervical region should be the standard initial diagnostic procedure and shows lucent streaks or bubbles of gas that outline mediastinal structures [11]. Other radiologic evidence of SPM includes thoracic and cervical subcutaneous emphysema (which is most apparent on lateral neck radiographs). The diagnosis should be confirmed with a chest computed tomography scan. Gastrografin esophagogram and bronchoscopy should be performed if there is suspicion of esophageal and tracheobronchial tree rupture.

Uncomplicated SPM is managed conservatively with analgesia, rest, and avoidance of maneuvers that increase pulmonary pressure (Valsalva or forced expiration) [7, 15]. The complications of SPM include pneumothorax with pneumomediastinum (requires chest tube), esophageal perforation with secondary pneumomediastinum (requires intensive medical and surgical management), tension pneumomediastinum (requires limited mediastinotomy), and pneumopericardium (requires surveillance for pericardial tamponade) [9, 10, 12].

## 4. Conclusion

SPM is a rare presentation of EVALI. To our knowledge, this is the first reported case of spontaneous pneumothorax in a healthy young man with daily vaping with concomitant influenza infection. Treatment of uncomplicated SPM is supportive consists of analgesia, oxygen therapy, rest, and avoidance of maneuvers that increase pulmonary pressure. Most patients recover without sequelae within a few days, and recurrence is rare.
